# Tools to develop antibiotic combinations that target drug tolerance in *Mycobacterium tuberculosis*

**DOI:** 10.3389/fcimb.2022.1085946

**Published:** 2023-01-06

**Authors:** Talia Greenstein, Bree B. Aldridge

**Affiliations:** ^1^ Department of Molecular Biology and Microbiology, Tufts University School of Medicine, Boston, MA, United States; ^2^ Graduate School of Biomedical Sciences, Tufts University School of Medicine, Boston, MA, United States; ^3^ Stuart B. Levy Center for Integrated Management of Antimicrobial Resistance, Boston, MA, United States; ^4^ Department of Biomedical Engineering, Tufts University School of Engineering, Medford, MA, United States

**Keywords:** tuberculosis, drug combinations, drug tolerance, dormancy, pharmacodynamics (PD), pharmacokinetics (PK), drug interaction

## Abstract

Combination therapy is necessary to treat tuberculosis to decrease the rate of disease relapse and prevent the acquisition of drug resistance, and shorter regimens are urgently needed. The adaptation of *Mycobacterium tuberculosis* to various lesion microenvironments in infection induces various states of slow replication and non-replication and subsequent antibiotic tolerance. This non-heritable tolerance to treatment necessitates lengthy combination therapy. Therefore, it is critical to develop combination therapies that specifically target the different types of drug-tolerant cells in infection. As new tools to study drug combinations earlier in the drug development pipeline are being actively developed, we must consider how to best model the drug-tolerant cells to use these tools to design the best antibiotic combinations that target those cells and shorten tuberculosis therapy. In this review, we discuss the factors underlying types of drug tolerance, how combination therapy targets these populations of bacteria, and how drug tolerance is currently modeled for the development of tuberculosis multidrug therapy. We highlight areas for future studies to develop new tools that better model drug tolerance in tuberculosis infection specifically for combination therapy testing to bring the best drug regimens forward to the clinic.

## Introduction

Tuberculosis (TB) remains notoriously difficult to treat and, until the COVID-19 pandemic, was the leading cause of death by a single infectious agent, *Mycobacterium tuberculosis* (Mtb) (WHO, 2021). TB requires lengthy combination therapy to prevent the acquisition of heritable drug resistance [addressed by others in this collection, including (Bhagwat et al., 2022; Jones et al., 2022; Liebenberg et al., 2022)] and to effectively target inherent heterogeneity in infection that results in drug tolerance to prevent treatment failure and subsequent disease relapse (Fox et al., 1999; Kerantzas and Jacobs, 2017). Genotypic resistance is defined by the heritable ability to grow in the presence of high concentrations of antibiotics beyond the minimum inhibitory concentration (MIC) (Balaban et al., 2019). Antibiotic tolerance is defined as the non-heritable ability of bacteria to survive transient exposure to drugs at concentrations that would otherwise be lethal (Brauner et al., 2016; Balaban et al., 2019). The current regimen for drug-sensitive Mtb requires a 2-month intensive phase of rifampicin, isoniazid, pyrazinamide, and ethambutol followed by a four or seven-month continuation phase of rifampicin and isoniazid. The phase III clinical trial (“Study 31”) for a four-month regimen with rifapentine, moxifloxacin, isoniazid, and pyrazinamide recently demonstrated non-inferiority to the six-month standard of care and is now recommended for some patients with drug-sensitive TB (Dorman et al., 2021; Carr et al., 2022). This progress notwithstanding, shorter and more effective therapies are urgently needed for both drug-sensitive and particularly for drug-resistant TB.

**Figure 1:**
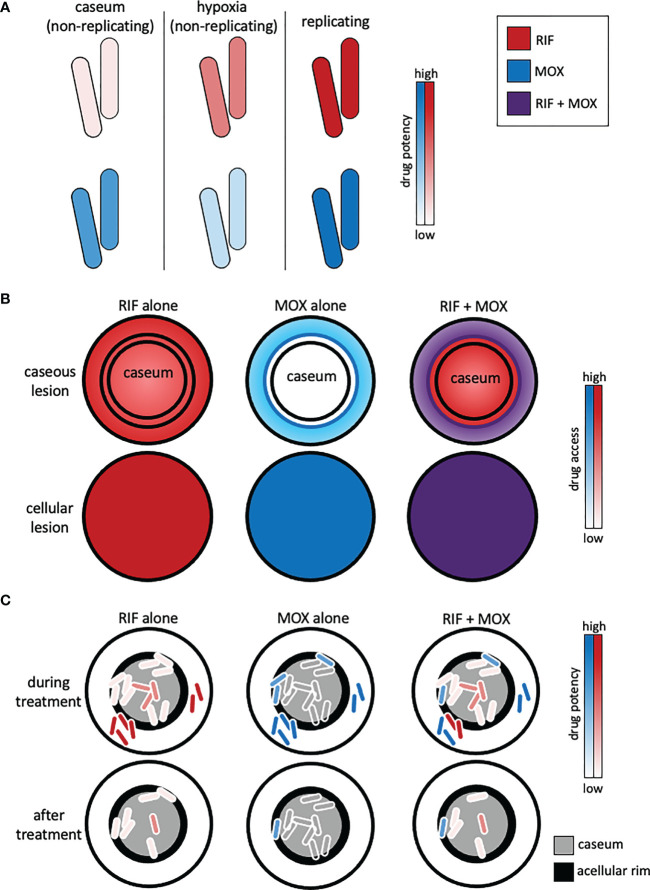
Representation of complex lesion pharmacokinetics and pharmacodynamics and how combination therapy must be optimized to reach different populations of drug-tolerant bacteria. **(A)** Drug pharmacodynamics are dependent on the bacterial state. Rifampicin (RIF) and moxifloxacin (MOX) exhibit different potencies against non-replicating intracaseum bacilli, non-replicating bacilli (e.g., induced by the Wayne hypoxia model), and replicating bacilli (e.g., in standard culture media). **(B)** Drug pharmacokinetics are lesion-dependent. Rifampicin (red) and moxifloxacin (blue) exhibit different levels of access to caseous lesions due to their drug-specific pharmacokinetic profiles. Inner concentric circles represent the acellular rim of caseous lesions. Drug combinations (e.g., RIF + MOX, purple) can overcome the pharmacokinetic limitations of single drugs. **(C)** Mtb that evade treatment and cause relapse are mainly found in the acellular rim (black) of caseum and in caseum. Drug combinations should be chosen to capitalize on pharmacokinetic and pharmacodynamic properties such that they access all the bacteria and kill the different types of drug-tolerant bacteria induced by the microenvironments in those locations.

A hallmark of TB pathogenesis is the formation of different lesion types that have varied structures and provide different microenvironmental conditions to resident Mtb (**Figure 1**). Mtb readily adapts to these different niches to withstand environmental stressors, often by slowing or halting replication and metabolic activity. These adaptations enable Mtb to tolerate drug treatment (Lenaerts et al., 2015; Sarathy and Dartois, 2020). Because the bacteria occupy separate niches with different environments, multiple states of drug tolerance exist together, influencing potential drug activity (pharmacodynamics) (Cadena et al., 2017). Furthermore, the physical structures of lesions vary, and drug penetration and accumulation in each lesion are dependent on the lesion structure and chemical properties of each drug (pharmacokinetics) (Prideaux et al., 2015; Sarathy et al., 2016) (**Figure 1**). Therefore, drug combinations with varied pharmacokinetic and pharmacodynamic properties are necessary to target the bacteria in all their locations.

More than twenty-five new TB drugs are at various stages across the developmental pipeline (newtbdrugs.org). There are 2,300 possible three-way combinations for twenty-five drugs, far too many to test *in vivo*. Single-drug responses may not be indicative of drug combination activity and potential to reduce relapse, and drugs may not behave in combination as they do alone due to drug interactions such as synergies and antagonisms. For example, pyrazinamide, a crucial member of the current standard of care regimen, is inactive against Mtb in many *in vitro* growth conditions and as a monotherapy *in vivo* (Steenken and Wolinsky, 1954; Cho et al., 2007; Lanoix et al., 2016). However, pyrazinamide contributes significant sterilizing activity when used for the first two months of treatment in combination with the other drugs in the standard of care (Fox et al., 1999). On the other hand, pyrazinamide unexpectedly exhibited antagonism when combined with rifampicin in a 14-day PET/CT clinical study (Xie et al., 2021). This finding was consistent with the prior evidence that the combination of rifampicin + pyrazinamide + isoniazid for the continuation phase of treatment displayed slightly higher relapse rates than rifampicin + isoniazid without pyrazinamide (East African- British medical research councils, 1973). Because combination therapy is necessary to treat TB and single-drug responses are not informative of combination activity or interactions, we need to be able to systematically screen and optimize combination therapies. However, it is logistically impossible to screen the entire drug combination space in animal infection models. Therefore, it is critical to be able to perform systematic combination screens using *in vitro* and computational tools that translate to animal and clinical outcomes. There is an urgent need for *in vitro* and computational tools that capture the complex pharmacodynamics (due to drug tolerance) and pharmacokinetics (due to lesion structure) in infection to design drug combinations that successfully target the bacteria that are hardest to access and kill.

Animal models have been a critical part of the preclinical development of drug combinations. Multiple models are used in the drug development pipeline to balance throughput, cost, and information gained. Heterogeneity across animal models and even within species complicates comparison across models. Mouse models have been used for decades; the most commonly used models until recently were the C57BL/6 and BALB/c models. These mice are genetically resistant to TB infection and make only cellular granulomas, thus presenting a major limitation in their ability to recapitulate human disease. The C3HeB/FeJ model has recently grown in popularity and use; this model is genetically susceptible to TB and makes both cellular and caseous granulomas, more similar to human disease (Driver et al., 2012). Larger animals (i.e., the New Zealand White rabbit, marmosets, cynomolgus and rhesus macaques) recapitulate human disease more closely than smaller animals but are logistically prohibitive for comprehensive early screens (Lenaerts et al., 2015). Given the massive combination landscape and logistical limitation of screening every combination in animal models, many *in vitro* models have been developed to mimic the different stressors encountered in lesions. The utility of these models in predicting animal and clinical outcomes is not always clear. Standard minimum inhibitory concentration assays using glucose-based media optimized for Mtb growth were the gold standard for a long time. However, it is well-established that glucose is not the primary carbon source for Mtb *in vivo*, and Pethe et al. demonstrated the importance of making measurements of drug response in more *in vivo*-like conditions when they identified a class of drugs with glycerol-specific activity against Mtb that was inactive *in vivo* (Pethe et al., 2010). This study also highlighted the importance of validating the predictive power of *in vitro* measurements of *in vivo* outcomes.

In this review, we will discuss how heterogeneous lesion microenvironments give rise to non-replication and subsequent drug tolerance, why this tolerance requires combination therapy for effective TB treatment, and the implications for screening assay design. We review the current state of *in vitro* and computational tools for drug screens and speculate on areas of improvement as they relate to drug tolerance and non-replication. New tools have been developed to allow for systematic study of higher-order drug combinations, including the DiaMOND methodology to reduce the number of measurements required in the higher-order combination screening space, a mathematical “dose” model to predict higher-order combination activity from drug pairs, and chemo-genomic and regulatory network models to predict drug interactions from single-drug transcriptomics (Peterson et al., 2016; Zimmer et al., 2016; Cokol et al., 2017; Ma et al., 2019). Beyond drug interactions, additional tools have been developed to consider single-drug and drug combination potency (Wooten et al., 2021; Li et al., 2022). Computational models have advanced to incorporate complex pharmacokinetics and pharmacodynamics to predict drug response outcomes informed by animal data and clinical data (Pienaar et al., 2017; Savic et al., 2017; Lyons, 2022). With all these developments, we have the opportunity to consider how to best utilize all these tools in the context of drug tolerance for the rational development of drug combinations that will be most effective in treating TB.

## Lesion microenvironments give rise to non-replication and subsequent drug tolerance

To identify drugs and combinations that will best target the bacteria that are most tolerant to treatment, it is critical to understand the underlying causes of the drug tolerance that is most difficult to treat, how we currently model environments that induce drug tolerance, and to fill the knowledge and technical gaps that will improve our current models. Heterogeneous lesions are the hallmark of TB infection, and these lesions present a variety of microenvironments to which Mtb adapts. TB granulomas are classically categorized as fibrotic, cellular/non-necrotic, caseous/necrotizing, or cavitating, each with distinct characteristics (Lenaerts et al., 2015). However, the reality of infection is more of a spectrum than discrete classes, and the microenvironmental components are not homogenous even within one lesion type (Barry et al., 2009). Mtb adaptation to these environments results in varied drug tolerance that requires combination therapy to treat effectively. A critical effect of many of the environmental components that the bacteria experience is slowing or halting replication. Non-replication is associated with extreme drug tolerance (Sarathy et al., 2018). In this section, we detail how different environments slow and halt Mtb growth, their influence on drug tolerance, and how they are modeled.

### Lipids

Lipids provide a crucial carbon source to Mtb in infection. Mtb has a remarkable capacity to metabolize different carbon sources (Wilburn et al., 2018). TB lesions are lipid-rich environments with varied lipid composition. Mtb induces the differentiation of infected macrophages into lipid-loaded foamy macrophages. These infected foam cells contain an abundance of triglycerides (TAG), cholesterol esters, and free cholesterol, and the composition is highly conserved across species (Guerrini et al., 2018). Fatty acid metabolism is required for Mtb to establish and maintain infection *in vivo* and cholesterol metabolism is required for Mtb persistence (Pandey and Sassetti, 2008; Marrero et al., 2010). Mtb co-metabolize both; they incorporate propionyl-CoA (an otherwise toxic byproduct of cholesterol metabolism) into their cell wall virulence lipids (e.g., PDIM) by utilizing host-derived long-chain fatty acids (LCFAs) to provide primers for their synthesis. Quinonez et al. showed that the accumulation of intermediates from the methylcitrate cycle, the pathway by which propionyl-CoA is formed, was associated with drug tolerance (Quinonez et al., 2022). It should be noted that though Mtb can co-metabolize cholesterol and many LCFAs, some LCFAs contribute to the slowing or arrest of Mtb growth (Lee et al., 2013; Rodriguez et al., 2014). Mtb resides in foamy macrophages in a non-replicating state (Peyron et al., 2008). As foamy macrophages become necrotic, they release the bacilli and their lipid-rich content, which accumulates to form lipid-rich caseum (Russell et al., 2009). Mtb is generally slowly replicating or non-replicating in caseum (Sarathy and Dartois, 2020). Mtb in lipid-rich conditions accumulate intracellular lipid inclusions (ILIs), which are associated with a dormant state and tolerance to rifampicin and isoniazid (Deb et al., 2009; Daniel et al., 2011). Intracaseum Mtb are remarkably tolerant to drugs; many antibiotics that are bactericidal in standard replicating conditions fail to sterilize caseum Mtb, and the concentrations of drugs required to kill Mtb are markedly higher (Sarathy et al., 2018).

Several *in vitro* models of Mtb lipid metabolism and drug response in lipid-rich conditions have been developed and utilized. As the importance of lipids in triggering Mtb “dormancy” (non-replication) has become more apparent, there has also been an increase in studies of Mtb transcriptome in lipid-rich conditions. These experiments have helped illuminate which lipids drive certain transcriptional and physiological responses (Rodriguez et al., 2014; Soto-Ramirez et al., 2017). For example, one study demonstrated that Mtb adaptation to a lipid-rich (LCFA-based) environment resulted in the overexpression of five genes in the DosR (dormancy) regulon. The transcriptional signature of Mtb adapted to LCFA was also compared against the signatures under hypoxia and starvation, and it was noted that the LCFA-adapted Mtb had more genes from the DosR regulon in common with Mtb under hypoxia than starvation (Rodriguez et al., 2014). These studies provide insight for choosing lipids to model Mtb adaptation to stressors encountered *in vivo*. A few high-throughput single-lipid models for drug screens have been developed, either short-chain fatty acid-based or cholesterol-based (Gold et al., 2015; Early et al., 2016; Larkins-Ford et al., 2021). One study identified anti-tubercular compounds with butyrate-specific activity that were not active against Mtb when cultured with glucose (Early et al., 2016). The caveat of single-lipid studies is that Mtb do not experience one lipid at a time, and it is plausible that outcomes from single-lipid studies might not translate across different lipids. Single-lipid models are typically growth models, but intracaseum Mtb, which experience a combination of many lipids, are slow or non-replicating. A combination of lipids, with or without additional stressors, might be more reflective of the environment(s) that Mtb experience *in vivo* and therefore provide a better model of lipid-driven dormancy and drug tolerance for drug combination studies. A notable such model is *ex vivo* rabbit caseum, where intracaseum Mtb from *ex vivo* rabbit caseum are treated with drugs. These bacilli were demonstrated to be non-replicating, but unlike simpler non-replicating models, the drug tolerance observed in these bacilli was more similar to that observed *in vivo*, making this a highly valuable tool for drug screens (Sarathy et al., 2018). High-throughput multi-stress models that achieve similar results to *ex vivo* caseum could therefore be an excellent tool for combination drug screens.

### pH

The pH in different lesions (and even within lesions) varies widely, from as low as 4.5 in the phagosomal compartment of the macrophages to 8 in caseum. The pH in caseum varies across animal models and from patient-to-patient, ranging from acidic to mildly basic (Lanoix et al., 2016; Kempker et al., 2017; Sarathy and Dartois, 2020). Recently, it was demonstrated that even within one lesion, the pH varies; a pH/Cl^-^ reporter strain was used to show that Mtb in infected C3HeB/FeJ mice experience more acidic pH at the lesion cuff than in the caseous core, and this correlated with reduced bacterial replication and increased antibiotic tolerance at the lesion cuff (Lavin and Tan, 2022). Mtb adaptation to acidic pH results in altered drug susceptibility (Baker et al., 2019). Mtb is viable at acidic pH but slows its growth with increasing acidity and halts growth entirely at pH 5.0 (Piddington et al., 2000; Baker et al., 2014). Notably, growth arrest at low pH is carbon source-specific; Mtb halts replication at pH 5.7 with glucose, glycerol, and TCA cycle intermediates but grows at pH 5.7 with host-associated carbon sources that function at the intersection of glycolysis and the TCA cycle (Baker et al., 2014). Mtb under acidic growth arrest are metabolically active (Baker and Abramovitch, 2018). Therefore, these cells are non-replicating but not necessarily “dormant” by the classical definition and may be metabolically or transcriptionally distinct from other populations of non-replicating Mtb induced by other microenvironments.

Though caseum is acidic in some lesions, the observation of neutral caseum in some lesions suggests that acidity is not the primary driving factor of non-replication and drug tolerance in caseum. A few *in vitro* models of Mtb adaptation to acidic pH have been developed for drug screens (Gold et al., 2015; de Miranda Silva et al., 2019; Early et al., 2019b; Larkins-Ford et al., 2021). One such model uses pH 4.5 to induce a non-replicating state and luminescent activity or a fluorescent reporter to measure viable bacteria (Early et al., 2019a; Early et al., 2019b). This assay was used to identify compounds with specific activity against non-replicating bacteria. Compounds with pH-dependent activity were also identified (Early et al., 2019a). A caveat of this model is that the acidic medium is low-nutrient. Therefore, it cannot be stated with certainty if the acidic pH was the primary driver of this particular non-replicating model, as starvation independently induces non-replication (Betts et al., 2002; Grant et al., 2013). A recent study demonstrated that LCFAs palmitic acid, oleic acid, and arachidonic acid enabled growth at pH 5.5, and supplementation of cholesterol to palmitic acid or oleic acid further enhanced this growth. Repeated supplementation of oleic acid permitted growth at pH as low as 4.5, albeit with decreasing growth rate (Gouzy et al., 2021). Therefore, in a low pH, low-nutrient model, the lack of nutrients potentially influences the bacterial ability to adapt to acidic pH and induces non-replication. A comparison of the transcriptome of different models of acid-adapted Mtb and other non-replication models may provide insight into the driving component of Mtb’s response in those models. Understanding the driving components of non-replication may be valuable in predicting drug tolerance profiles (e.g., how tolerant the bacteria are to a set of drugs). For example, if each non-replicative state induced by different conditions is transcriptionally distinct, will they exhibit unique drug tolerance profiles? Furthermore, is there a primary driver in multi-stress-induced non-replication that results in a similar transcriptome and drug tolerance profile for the multi-stress to that of the primary driver, or are multi-stress models of non-replication transcriptionally distinct from the single-stress models? This information could be used to predict the drug tolerance profile of bacteria in specific locations of a lesion based on the type of non-replication induced by the local environmental components.

### Ion and metal availability

Macrophage response to infection includes changes in ion flux, and Mtb has a variety of mechanisms to adapt to these responses (Neyrolles et al., 2015). Mtb responds to high chloride in a linked transcriptional response to acidic pH during phagosomal maturation, and Mtb potassium uptake is shown to play a role in host colonization (Tan et al., 2013; MacGilvary et al., 2019). Zinc-limited Mtb display decreased sensitivity to oxidative stress and some antibiotics (Dow et al., 2021). Zinc limitation signals Mtb to build alternative ribosomes (Prisic et al., 2015). These alternative ribosomes were shown to be essential for *M. smegmatis* growth in an iron-depleted environment (Chen et al., 2020). Mtb upregulate virulence factors in response to iron limitation (Rodriguez et al., 2022); in turn, iron starvation leads to the transition of Mtb to a non-replicative state and subsequent drug tolerance (Kurthkoti et al., 2017). Intracellular (macrophage) Mtb exhibit a similar transcriptional response to Mtb cultured in low-iron media, indicating Mtb experience a low-iron environment in the phagosome (Schnappinger et al., 2003). These different states may therefore represent a critical component of the host response to infection that should be considered in *in vitro* models.

Ion perturbation models are largely overlooked for drug screens; ion studies tend to be mechanistic or study effects on host colonization rather than drug susceptibility. However, a recent macrophage infection drug screen demonstrated that one of the hit compounds limited Mtb’s access to iron by acting as an iron chelator. Mtb also has a greater dependency on iron when cultured with cholesterol as the sole carbon source, linking iron uptake and cholesterol metabolism (Theriault et al., 2022). Another recent study demonstrated that iron levels play a role in modulating transcriptional responses to growth arrest when Mtb transition from exponential growth to stationary phase (Alebouyeh et al., 2022). Iron uptake’s role in non-replication and the influence of iron availability on drug tolerance warrant further investigation and incorporation into drug response screens.

### Oxygenation

Hypoxia has been recognized as an important feature within some granulomas. Caseous necrotic granulomas in guinea pigs, rabbits, and non-human primates were all demonstrated to be hypoxic (Via et al., 2008). C57BL/6 and BALB/c mice only develop cellular lesions, which are not hypoxic, but C3HeB/FeJ mice develop hypoxic caseous necrotic lesions (Harper et al., 2012). Mtb exposure to hypoxia induces expression of the Mtb dormancy survival regulator *dosR*. Hypoxia induces dormancy by the more classical definition of non-replication and decreased cellular functions, including DNA and protein synthesis (Sherman et al., 2001; Rao et al., 2008). Mtb is markedly more tolerant to drugs in an anaerobic environment relative to aerobic (Cho et al., 2007).

One of the first major models of Mtb survival in hypoxia is the Wayne model, in which Mtb descend into a hypoxic environment in a controlled, gradual manner. They describe two distinct states of non-replicating persistence, NRP1 and NRP2, each with distinct drug-dependent drug tolerance profiles (Wayne and Hayes, 1996). While both are non-replicating, they have distinct transcriptional profiles (Muttucumaru et al., 2004). Though this model is not amenable to high-throughput screens, it highlights how Mtb survives using transcriptionally distinct states of non-replication and how this translates to different types of drug tolerance. Abramovitch and colleagues utilize another model of gradual descent into hypoxia in which Mtb grows in multi-well plates for six days, resulting in oxygen consumption and promoting hypoxic conditions at the bottom of the wells (Zheng et al., 2017). The low-oxygen-recovery assay (LORA) is a drug screen assay where drug-treated autoluminescent bacteria are placed under anaerobic conditions for ten days and recovered with oxygen for 28 hours, after which luminescence is measured (Cho et al., 2007). The LORA can be used to identify drugs with activity against non-replicating bacteria. This assay does not specifically distinguish between NRP1 and NRP2; based on the timescale of the assay, it is a model of NRP1. A caveat of the model is that there could be drug carryover during the recovery phase. Given the evidence that non-replicating bacteria are more drug-tolerant than replicating, drug carryover into the recovery phase could potentially confound the results. This might explain the noted discrepancies between luminescence assay-based minimum inhibitory concentrations (MICs) and colony-forming unit (CFU)-based MICs (Cho et al., 2007).

It is unlikely that Mtb experience sudden anaerobiosis *in vivo*; rather, access to oxygen likely decreases gradually. Sudden anaerobiosis is lethal to Mtb cultures. The Wayne and LORA models both create anaerobic conditions gradually, allowing Mtb to adapt and halt replication. Mtb can respire nitrate in the absence of oxygen and nitrate enhances Mtb survival under anaerobiosis (Sohaskey, 2008). Therefore, some hypoxic models use sodium nitrate as an alternate electron acceptor for Mtb. Gold et al. developed a multi-stress model that includes a lipid carbon source (butyrate), hypoxia, sodium nitrate, and acidic pH (5.5). This combination of stressors induces non-replication. The multi-stress model is unique in that three of the media components are independent drivers of dormancy (hypoxia, reactive nitrogen intermediates, and acidic pH without starvation). The assay compares replicating and non-replicating bacteria and distinguishes bacteriostatic and bactericidal compounds. In lieu of plating and counting colony-forming units to quantify drug effect, which is too resource-intensive, this model was paired with the charcoal agar resazurin assay (CARA), wherein the drug-treated bacteria are transferred to agar with activated charcoal in multi-well format for outgrowth for seven days, and then fluorescence is measured. The activated charcoal serves to inactivate any carryover drug (Gold et al., 2015). The CARA is a useful method for measuring drug activity against non-replicating bacteria in a high throughput manner. The multi-stress model developed by Gold et al. was recently modified to use sodium nitrate and hypoxia in a lipid-rich environment to induce dormancy for drug combination screening (Larkins-Ford et al., 2022).

Many interesting questions remain unanswered: is there a distinction between combination-stressor-induced dormancy versus single-component-induced dormancy as they relate to drug tolerance? If one stressor (i.e., hypoxia) can induce multiple states of non-replicating drug tolerance (i.e., NRP1 and NRP2), then it is plausible that multiple stressors could, as well. Understanding the different transcriptomic and drug response profiles of different dormancy/non-replication states is key to identifying which are most important to model for drug combination screens. Mtb can survive in a dormant state for a remarkably long time; one study demonstrated that after one year of dormancy, Mtb’s proteome remained similar to the proteome of 4-month dormant bacteria (Trutneva et al., 2020). Though many different models of non-replication have been developed, the time of bacterial exposure to the conditions is typically short (<= seven days), and an in-depth analysis of the relationship between time spent in a non-replicating or dormant state and drug tolerance is lacking. Furthermore, does the method of induction of non-replication influence the rate at which Mtb can resume replication from a non-replicating state? Soto-Ramirez et al. have shown differential gene expression and different rates of change in gene expression upon re-oxygenation from NRP1 and NRP2 states dependent on lipid carbon source, indicating that the growth environment may play a role in when and how Mtb exits a non-replicative state (Soto-Ramirez et al., 2017). When caseous lesions cavitate, the bacteria rapidly switch from a hypoxic environment to high oxygenation, allowing them to exit dormancy and resume replication. Mtb’s ability to rapidly recover from non-replication therefore influences its survival.

## Combination therapy is key to preventing relapse: The connection to drug tolerance

Combination therapy’s superiority over monotherapy in bactericidal activity and preventing relapse is well-established; however, drug screens and animal and clinical studies typically focus on outcomes and rarely investigate and understand the relationship between this superiority and targeting drug tolerance. Early studies in the development of the current standard of care (isoniazid + rifampicin + pyrazinamide + ethambutol, or HRZE) found that the addition of rifampicin or pyrazinamide to the treatment regimen with streptomycin and isoniazid reduced disease relapse and including both allowed for shorter treatment duration (Fox et al., 1999). Though it was initially thought that this success was due to drug synergy (East African- British medical research councils, 1974), it was ultimately attributed to activity against semi-dormant bacteria (Dickinson and Mitchison, 1981). This was demonstrated using *in vitro* studies of isoniazid- or rifampicin-treated Mtb whose growth rate was stalled by decreasing incubation temperature or culture in acidic conditions. Short exposure to optimal growth conditions resulted in short recovery bursts, during which rifampicin had greater bactericidal activity than isoniazid, suggesting that rifampicin is better at killing these bacilli that are semi-dormant, that is, dormant much of the time with occasional metabolic bursts. Pyrazinamide has also been demonstrated specifically to target dormant (non-replicating) bacilli and loses its sterilizing activity when metabolic activity resumes (Hu et al., 2006). Therefore, using multiple drugs in combination may reduce the incidence of relapse by targeting multiple bacterial states in infection. In support of this, Mitchison and colleagues used a guinea pig model and showed that the addition of rifampicin to isoniazid or isoniazid + ethambutol did not increase the bactericidal activity of the combination but reduced relapse, suggesting that the success of the combination was not in additional killing, but in killing particular cells that would have otherwise caused relapse (Dickinson and Mitchison, 1976). Walter et al. proposed a ribosomal RNA synthesis ratio as a metric to distinguish sterilizing and non-sterilizing drugs and drug combinations and demonstrated its utility both *in vitro* and in the relapsing mouse model (Walter et al., 2021). This method could be used to provide molecular insight into how sterilizing combinations modulate cellular processes in subpopulations of drug-tolerant Mtb. Furthermore, the combination of the ribosomal RNA synthesis ratio with CFU measurement in the mouse model was recently found to be more informative of treatment-shortening potential than either metric alone (Dide-Agossou et al., 2022). A combination of pharmacodynamic markers could enhance our understanding of how combination therapy targets drug-tolerant cells both *in vitro* and *in vivo*.

Successful treatment of TB requires killing the bacteria that would otherwise remain to cause relapse. These cells may reside in different lesions and therefore exhibit different types of drug tolerance due to adaptation to different microenvironments. Combination therapy with multiple modes of action offers greater potential over monotherapy to kill multiple types of drug tolerance, and the best combinations will be those that successfully target different types of drug tolerance. Though this has not been demonstrated systematically, empirical evidence supports this concept: bedaquiline and pretomanid, which have performed remarkably well in both animal and clinical studies and have been approved in combination with linezolid for MDR-TB (NiX-TB) (Conradie et al., 2020), target both replicating and non-replicating bacteria, and have been shown to be particularly potent against non-replicating bacteria induced by different microenvironmental conditions (Cho et al., 2007; Koul et al., 2008; Tasneen et al., 2011; Gold et al., 2015). Their superior sterilizing performance could be attributed to their ability to target multiple types of drug tolerance. Recently, a four-month regimen with rifapentine and moxifloxacin was found to be non-inferior to the standard of care (“Study 31”) (Dorman et al., 2021). In preclinical studies, moxifloxacin exhibited potent early bactericidal activity in treatment (Nuermberger et al., 2004). Moxifloxacin’s potent activity against both replicating and non-replicating Mtb may contribute to this more rapid clearance. Taken together, the evidence suggests that combination therapy is required to reduce relapse, and the most effective regimens will include drugs that target multiple types of drug tolerance.

## Lesion structure influences drug access: Consideration in drug screens

Heterogeneous lesions also result in varied drug access, absorption, and metabolism (i.e., pharmacokinetics) dependent on lesion structure, which drives the necessity of combination therapy to ensure that one or more of the drugs can access all the different locations and be effective in those locations (**Figure 1**). MALDI mass spectrometry imaging (MSI) has been used to show how different drugs penetrate caseum *in vivo* (Sarathy et al., 2016). Pyrazinamide and isoniazid penetrate fairly evenly, but bedaquiline, which is highly lipophilic, binds intracellular lipids and caseum macromolecules and penetrates caseum very poorly (Sarathy et al., 2016; Greenwood et al., 2019). Clofazimine, which is highly effective against Mtb in BALB/c mice (which make exclusively cellular lesions), is relatively ineffective against Mtb in C3HeB/FeJ mice (which develop caseous necrotic lesions) (Irwin et al., 2014). Pharmacokinetic modeling demonstrated that clofazimine accumulates in cellular layers and does not diffuse into necrotic foci, explaining the lack of efficacy in the C3HeB/FeJ mice (Prideaux et al., 2015). Therefore, modeling lesion drug access to ensure that sufficient drug(s) reach the different types of drug tolerance in all their locations is important for the design of effective combinations.

Pharmacokinetics are also important when considering drug interactions; if an antagonistic combination of drugs do not act in the same location, the antagonism may not be realized. Lesion-specific and caseum-specific pharmacokinetics are studied using a combination of MSI *in vivo*, *ex vivo* rabbit caseum, and *in vitro* caseum surrogate derived from foamy THP-1 macrophages (Sarathy et al., 2016). Lesion pharmacokinetics are also modeled using computational models, typically informed by *in vivo* data (Kjellsson et al., 2012; Prideaux et al., 2015). Most pharmacokinetic/dynamic (PK/PD) models do not consider drug interactions. Recently INDIGO-MTB, a computational tool to predict drug interactions using transcriptomics, was integrated with GranSim, a multi-scale model of tuberculosis granuloma formation. GranSim incorporates host immunity, Mtb growth dynamics, and drug PK/PD into one computational framework that describes interactions between these entities through space and time in granuloma evolution (Cicchese et al., 2021). Expanding this and other models to consider drug combinations, drug interactions, varied drug tolerance, and lesion-specific pharmacokinetics could provide invaluable information for the development and improvement of effective drug combinations.

## Computational modeling to link *in vitro* models with animal and clinical outcomes

Though many lesion microenvironmental components are thought to affect drug susceptibility in some capacity, it may be that we do not need to model every single one to capture the drug tolerance that causes disease relapse, i.e., the bacteria that are hardest to treat. Instead, we should identify which of these environmental niches and resultant bacterial states represent the bacteria that are most difficult to treat. Using a guinea pig model, Lenaerts and colleagues showed that the bacteria that remained after treatment were extracellular, primarily in the hypoxic acellular rim of caseous necrosis (Lenaerts et al., 2007). A marmoset study showed that the difference between sterilizing and non-sterilizing regimens was rapid clearance of cavitating lesions by the sterilizing regimen, indicating that drug combinations must target the cavitating lesion for a better outcome (Via et al., 2015). Given the complexity of the tuberculosis heterogeneity, computational models are a critical tool to link *in vitro* drug responses to such treatment outcomes in animals and the clinic. Recently Larkins-Ford et al. used a machine learning approach to predict relapse outcomes in the BALB/c mouse model from a suite of drug combination potency and interaction measures made in a variety of growth conditions to model the different environments in TB lesions (Larkins-Ford et al., 2021). This study demonstrated that *in vitro* measurements of response to drug combinations made in a subset of growth conditions (as a “sum of parts”) could be predictive of *in vivo* outcomes. Some sets of conditions were more predictive of *in vivo* response than others, thereby identifying validated sets of *in vitro* models as suitable for drug combination screening. We have yet to understand whether or not measurement in more complex growth conditions that combine these “parts” into one condition (i.e., a multi-stress model) will be more predictive than the sum of parts approach. Improving *in vitro* models to capture the environments that result in drug tolerance that enables the bacteria to survive beyond treatment and correlating response in these models to *in vivo* outcomes is a clear next step. Computational and mathematical modeling offer the advantage that they are inherently optimal for iterative learning and can leverage *in vivo* outcome data.

To get the best prediction from computational models, the input from *in vitro* models must be optimized and streamlined. Several tools have been developed to improve the quality and utility of metrics from *in vitro* models of drug combination activity (summarized in **Table 1**). Diagonal measurement of n-way drug interactions (DiaMOND) is a method to measure drug interactions using only a fraction of the drug-dose combination matrix (“checkerboard”), which is logistically prohibitive to measure for higher-order of systematic studies (Cokol et al., 2017). DiaMOND measures dose response curves, enabling the collection of additional metrics beyond drug interaction, including combination potency metrics. MuSyC is another framework that calculates drug interactions and distinguishes between different types of combination effects (whether we consider dose or efficacy to evaluate drug synergy) to overcome conflicting assumptions of the widely used Loewe Additivity and Bliss Independence principles and to account for drug interactions and combination potencies (Wooten et al., 2021). This widening of combination effects from only measuring traditional synergy may be important in our ability to develop predictive models of *in vivo* outcomes from *in vitro* data. Larkins-Ford et al. recently demonstrated that combination potency metrics were important to accurately predict treatment outcomes in mouse models (Larkins-Ford et al., 2021; Larkins-Ford et al., 2022). Additional modeling techniques utilize pairwise drug response measurements to predict high-order drug interactions (Zimmer et al., 2016; Katzir et al., 2019) and *in vivo* treatment outcomes (Larkins-Ford et al., 2022) as a path to reduce the number of measurements required to study the increasingly large drug combination space and still obtain informative metrics beyond traditional drug interactions. Other new computational tools incorporate pathway-specific effects of drug action to predict drug interactions (Peterson et al., 2016). INDIGO (inferring drug interactions using chemo-genomics and orthology), for example, uses transcriptomic data from single-drug responses to predict drug interactions (Chandrasekaran et al., 2016; Ma et al., 2019). These integrated molecular approaches may help us understand the pathways underlying drug tolerance and combination drug response.

**Table 1:** Summary of mathematical frameworks and computational tools used to study drug combinations and PK/PD in TB.

Method	Application	Advantage to pipeline	Limitations
Dose model (Zimmer et al., 2016; Katzir et al., 2019)	Mathematical model to predict higher-order *in vitro* drug combination response from pairwise data	★ Reduces the number of combination measurements needed by predicting higher-order measurements above pairwise	• Predicts *in vitro* values (not *in vivo*) •Not compatible with drugs that cannot achieve complete inhibition or killing
INDIGO (Chandrasekaran et al., 2016; Ma et al., 2019; Cicchese et al., 2021)	Machine learning model to predict drug interactions using single-drug transcriptomic data	★ Reduces the number of combination measurements needed ⧠ Provides molecular insights ❖ predicted interactions shown to correlate with clinical efficacy ❖ Used with GranSim to predict influence of drug interaction on bacterial killing rate in granulomas and correlate to clinical efficacy	• Does not account for dose response or pharmacokinetics • Predicts only drug interaction metrics • Merger of gene expression data from different sources may require batch normalization
DiaMOND (Cokol et al., 2017; Larkins-Ford et al., 2021)	Methodology to reduce the number of *in vitro* measurements necessary to capture the drug combination checkerboard space	★ Increased efficiency in *in vitro* measurements enables more combinations and conditions to be screened ⧠ Combination dose response offers multiple usable and interpretable metrics for prediction of *in vivo*/clinical outcomes ❖ Used in machine learning models to predict treatment outcomes of large numbers of combinations in preclinical models	• Ideal approximation of synergy requires equally potent concentrations of drug in combination; deviation from equipotency compromises accuracy • Equally potent concentrations of drugs in combination do not reflect pharmacokinetics • This geometric approximation will not be accurate for very asymmetric drug interaction
Hollow fiber model (Gumbo et al., 2004; Gumbo et al., 2015)	*In vitro* tool to model and measure PK/PD combined with computational modeling	⧠ Experimental approach incorporates PK *in vitro*, allowing for *in vitro* regimen design ❖ Used in Monte Carlo simulations to predict optimal doses of drugs ❖ Used to predict outcome in clinical patients from *in vitro* data	• Low-medium throughput • Does not model components of the immune response
GranSim (Fallahi-Sichani et al., 2011; Pienaar et al., 2017; Cicchese et al., 2021)	Multi-scale spatial-temporal model of Mtb-immune cell dynamics in granuloma formation and resolution with drug treatment	★ Parameters easily changed for new simulations (e.g., to modulate specific cytokine production) ❖ Outcome combines granuloma immune contribution with drug response ❖ Used to predict granuloma outcomes in response to drug treatment (e.g., bacterial burden, time to sterilization) ❖ Tool to design and optimize regimens for preclinical models	• Running agent-based models is computationally intensive • Low-medium throughput
PK/PD modeling (Savic et al., 2017; Strydom et al., 2019; Fors et al., 2020)	Model to analyze drug exposure-response relationship in clinical population	❖ Provides clinical dosing strategy based on treatment-shortening potential (used for this purpose for Study 31) ❖ Outcome combines host immune contribution with drug response ❖ Predicts clinical population and individual outcomes ❖ Tool to design and optimize regimens for preclinical models and clinical trials	• PK/PD models use animal data inputs to set model parameters, therefore relying on assumptions that scaling between species and host immune response across species are equivalent • Low-medium throughput

Symbols under “Advantage to pipeline” represent advantage class: ⧠ novel combination effects (i.e., metrics) ★ increases efficiency of combination measurement ❖ capable of or used for prediction of *in vivo* or clinical outcomes.

More complex, mechanistic computational models integrate host-pathogen interactions, pharmacodynamics, and pharmacokinetics (summarized in **Table 1**) (Ernest et al., 2021). GranSim is a multi-scale, agent-based model that captures the temporal and spatial dynamics of immune cell activity, bacterial growth, and bacterial killing by drugs in different lesion types, informed by pharmacokinetic and pharmacodynamic data from non-human primates and rabbits. These dynamics are captured at the molecular, scale, and whole lesion scales. As new *in vivo* data are acquired, the model may be updated. GranSim has demonstrated how changes in cytokines influence early infection and lesion formation (Fallahi-Sichani et al., 2011; Wong et al., 2020). GranSim can also be integrated with other computational frameworks, including INDIGO-MTB and a constraint-based model (CBM) that predicts Mtb metabolism and growth. GranSim-CBM is used to predict how environmental influence on Mtb metabolism and growth influences granuloma development and outcomes (Pienaar et al., 2016). The hollow fiber system model of tuberculosis is an *in vitro* drug development tool to optimize drug regimens and dose selection to maximize drug or combination efficacy and minimize the emergence of genotypic resistance. The *in vitro* system has a pharmacodynamic compartment that houses the bacteria and a pharmacokinetic compartment with semipermeable hollow fibers that allow drugs to diffuse to the pharmacodynamic compartment in a manner that mimics the appropriate concentration-time profile for the drugs. The results of the *in vitro* experiments are used in Monte Carlo simulations to predict outcomes in clinical populations (Gumbo et al., 2004; Gumbo et al., 2015). Another PK/PD model incorporates human clinical data to simulate the outcome of various multi-drug regimen scenarios (available at http://www.saviclab.org/systems-tb/) (Fors et al., 2020). Savic and colleagues have developed population PK/PD models that were used to determine the optimal dose of rifapentine with the most potential to shorten the duration of treatment based on outcomes from phase II clinical trials (Savic et al., 2017). This information was used to inform the dosing strategy for the phase III clinical trial for Study 31, which was found noninferior to the 6-month standard of care (Dorman et al., 2021). Incorporating validated *in vitro* model data that capture the drug tolerance *in vivo* in PK/PD models offers the potential to predict treatment outcomes at the granuloma level and to fine-tune and optimize regimens to target the bacteria that would otherwise withstand treatment either due to drug tolerance or poor drug access and cause relapse.

Though the field of computational models and mathematical frameworks for measuring combination drug response has seen tremendous progress and innovation, gaps remain. Tools like INDIGO-MTB and the dose model enable the prediction of higher-order interactions from single-drug transcriptomics or pairwise drug interactions and raise the question of the necessity of higher-order combination screens. Recently Larkins-Ford et al. demonstrated that synergy and potency metrics derived from pairwise combination measurements could be used to build successful higher-order combinations (Larkins-Ford et al., 2022). Streamlining predictive metrics of treatment outcomes (both animal and clinical) and the definition of “systematic” (is pairwise sufficient or is higher-order necessary)? is important to improve the *in vitro* data that will be used in modeling. There is also a need for a system to report failed regimens to improve computational models. Understanding why regimens failed will help inform model parameters to identify other combinations that might also fail.

## Looking forward: The future of TB combination therapy and models of drug tolerance

The development of improved, streamlined *in vitro* models that inform computational models is not the end of the line. Additional considerations that may improve models include single-cell heterogeneity and strain-to-strain differences. For example, innate growth and metabolic differences among closely related bacilli create subpopulations that are differentially susceptible to drug treatment (Aldridge et al., 2012; Manina et al., 2015; Rego et al., 2017; Shee et al., 2022). Antibiotic-stressed, replicating mycobacteria display drug tolerance at the single-cell level; antibiotic-induced tolerance and tolerance in replicating Mtb warrant further study (Wakamoto et al., 2013; Zhu et al., 2018). It is unknown if and how much single-cell heterogeneity contributes to the subpopulations of bacteria in humans that survive beyond treatment to cause relapse. Once we have improved and validated *in vitro* models of non-replicating Mtb that survive beyond treatment, single-cell studies can be used to determine if certain cells exhibit identifiable transcriptomic or morphological markers that pre-dispose them to greater drug tolerance. We might also consider the effects of resistance mutations on drug combination activity. Schrader et al. showed that mutations that arise due to exposure to one antibiotic can cause multiform antibiotic resistance, i.e., different types of drug tolerance that may not be heritable (Schrader et al., 2021).

**Figure 2:**
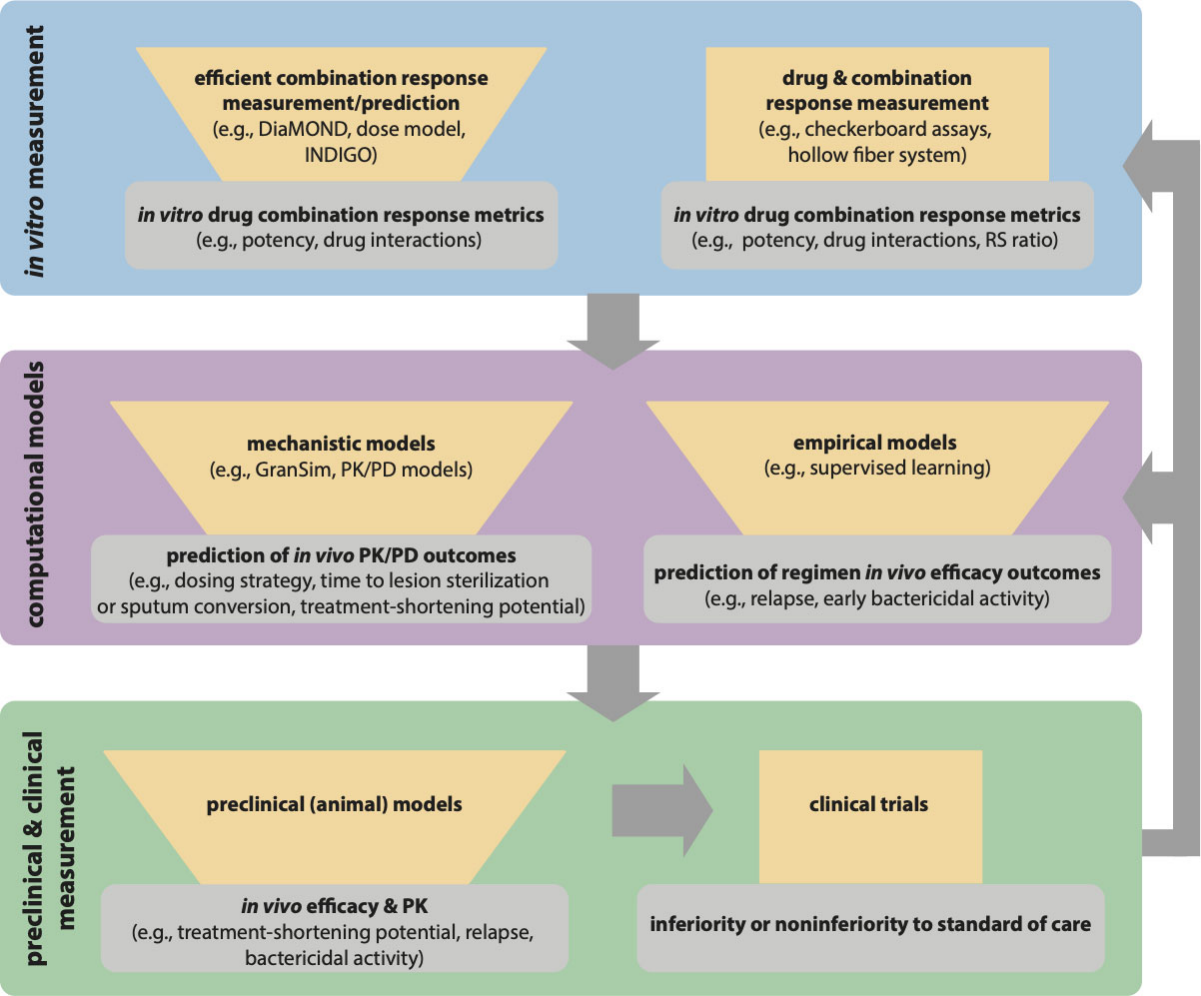
Paradigm for iterative modeling of drug combinations using *in vitro* and computational tools to reduce the drug combination space for *in vivo* testing and clinical trials. Each iterative step of drug combination design should reduce the number of combinations or regimens to test in the next step (represented with funnels). The tools in the *in vitro* exploration space (blue) allow for exhaustive combination screens while requiring fewer measurements. Specialized tools like the hollow fiber system allow for *in vitro* PK/PD exploration. The output of *in vitro* models is input into computational models (purple) for empirical prediction of *in vivo* efficacy and used with *in vivo* PK study data in mechanistic predictions of *in vivo* PK/PD outcomes. The best combinations from model predictions can then go back to the *in vitro* space (blue) for expanded testing (e.g., with different Mtb strains, different culture conditions), and the prioritized combinations from expanded *in vitro* testing would be input again through computational models (gray arrows). The best regimens from model predictions are tested in preclinical models (green), and the best regimens from preclinical models can go back to *in vitro* exploration and computational models for further refinement of the models and experimental design (gray arrows). Iterative improvement of regimens allows for optimized combinations to go to clinical trials.

Effective targeting of drug-tolerant Mtb will also require that we understand how Mtb lineage contributes to different manifestations of tolerance. Different Mtb strains exhibit differences in virulence, adaptation to environmental stressors, and drug tolerance (De Groote et al., 2012; Tizzano et al., 2021). Using genome-wide association studies (GWAS), Hicks et al. identified SNPs in clinical strains of the Beijing lineage associated with propionate metabolism that conferred drug tolerance to certain drugs (Hicks et al., 2018). Recently Li et al. utilized CRISPRi technology to quantify the expression of Mtb genes and bacterial fitness in the presence of different drugs and discovered genetic mechanisms of intrinsic drug tolerance. Upon comparing this data against genomics of Mtb clinical isolates, strain-dependent mutations were discovered that conferred specific drug susceptibility (Li et al., 2022). These studies offer the possibility to tailor drug combinations to Mtb lineage based on genetic differences across lineages. Another recent study showed that the *resR* transcriptional regulator is a frequent target of positive selection for mutations and that strains with these mutations exhibit antibiotic “resilience,” which describes the potential for antibiotic tolerance and genetic resistance. The bacilli harboring these mutations exhibited more rapid post-antibiotic recovery than wild-type cells (Liu et al., 2022). This new finding demonstrates that we must better understand the various strategies Mtb uses to recover from drug treatment beyond standard definitions of non-replication and dormancy so that we can design combination therapies that target these subpopulations. To ensure that drug combinations adequately target tolerance across lineages and resilient subpopulations, multiple (clinical and laboratory) strains could be used in *in vitro* models for combination drug screens. We propose an iterative learning process (**Figure 2**) to develop improved drug combinations where *in vitro* models of non-replicating-induced drug tolerance are used to screen drug combinations. Streamlined drug interaction and potency metrics would be used as input for computational models of complex PK/PD to identify better drug combinations to target drug-tolerant Mtb. Computational models should then identify potential populations that could cause relapse and then loop back to the *in vitro* models to redesign the combinations to better target those populations. Multiple strains could then be tested and used as additional input in the computational model so that the best regimens that target drug tolerance across lesions and strains are carried forward to test in animals and the clinic.

## Concluding remarks

TB requires lengthy multidrug treatment due to populations of drug-tolerant bacteria that arise from the heterogeneity of infection. Complex lesion structure and microenvironments induce multiple types of non-replicating bacterial populations with differential drug susceptibility and variable drug access. Combination therapy is required to target these populations in all their locations, and better tools for *in vitro* screens are urgently needed. The ability to screen combinations systematically will enable us to consider the best combinations that will target drug-tolerant cells in a data-driven manner. Incorporating these data in lesion-scale PK/PD models that simulate which bacteria evade kill will also help inform which niches in the lesions induce the drug tolerance that results in relapse. Understanding which bacterial subpopulations remain will inform better choices of compounds to target those types of tolerance. Those drugs can then be included in systematic combination screens to design optimized combinations that target the types of drug-tolerant bacteria that evade kill. To achieve this goal, we must fill the knowledge gaps delineated in this review about the drug-tolerant non-replicating populations, streamline measurements from combinations screens, and optimize computational models of PK/PD to account for drug combinations and multiple types of drug tolerance.

## Author contributions

Writing and editing: TG and BA. All authors contributed to the article and approved the submitted version.

## References

[B1] Aldridge B. B. Fernandez-Suarez M. Heller D. Ambravaneswaran V. Irimia D. Toner M. . (2012). Asymmetry and aging of mycobacterial cells lead to variable growth and antibiotic susceptibility. Science 335 (6064), 100–104. doi: 10.1126/science.1216166 22174129 PMC3397429

[B2] Alebouyeh S. Cardenas-Pestana J. A. Vazquez L. Prados-Rosales R. Del Portillo P. Sanz J. . (2022). Iron deprivation enhances transcriptional responses to *in vitro* growth arrest of mycobacterium tuberculosis. Front. Microbiol. 13. doi: 10.3389/fmicb.2022.956602 PMC9577196 36267176

[B3] Baker J. J. Abramovitch R. B. (2018). Genetic and metabolic regulation of mycobacterium tuberculosis acid growth arrest. Sci. Rep. 8 (1), 4168. doi: 10.1038/s41598-018-22343-4 29520087 PMC5843633

[B4] Baker J. J. Dechow S. J. Abramovitch R. B. (2019). Acid fasting: Modulation of mycobacterium tuberculosis metabolism at acidic pH. Trends Microbiol. 27 (11), 942–953. doi: 10.1016/j.tim.2019.06.005 31324436 PMC6800632

[B5] Baker J. J. Johnson B. K. Abramovitch R. B. (2014). Slow growth of mycobacterium tuberculosis at acidic pH is regulated by phoPR and host-associated carbon sources. Mol. Microbiol. 94 (1), 56–69. doi: 10.1111/mmi.12688 24975990 PMC4177513

[B6] Balaban N. Q. Helaine S. Lewis K. Ackermann M. Aldridge B. Andersson D. I. . (2019). Definitions and guidelines for research on antibiotic persistence. Nat. Rev. Microbiol. 17 (7), 441–448. doi: 10.1038/s41579-019-0196-3 30980069 PMC7136161

[B7] Barry C. E. 3rd Boshoff H. I. Dartois V. Dick T. Ehrt S. Flynn J. . (2009). The spectrum of latent tuberculosis: rethinking the biology and intervention strategies. Nat. Rev. Microbiol. 7 (12), 845–855. doi: 10.1038/nrmicro2236 19855401 PMC4144869

[B8] Betts J. C. Lukey P. T. Robb L. C. McAdam R. A. Duncan K. (2002). Evaluation of a nutrient starvation model of mycobacterium tuberculosis persistence by gene and protein expression profiling. Mol. Microbiol. 43 (3), 717–731. doi: 10.1046/j.1365-2958.2002.02779.x 11929527

[B9] Bhagwat A. Deshpande A. Parish T. (2022). How mycobacterium tuberculosis drug resistance has shaped anti-tubercular drug discovery. Front. Cell Infect. Microbiol. 12. doi: 10.3389/fcimb.2022.974101 PMC9500310 36159638

[B10] Brauner A. Fridman O. Gefen O. Balaban N. Q. (2016). Distinguishing between resistance, tolerance and persistence to antibiotic treatment. Nat. Rev. Microbiol. 14 (5), 320–330. doi: 10.1038/nrmicro.2016.34 27080241

[B11] Cadena A. M. Fortune S. M. Flynn J. L. (2017). Heterogeneity in tuberculosis. Nat. Rev. Immunol. 17 (11), 691–702. doi: 10.1038/nri.2017.69 28736436 PMC6247113

[B12] Carr W. Kurbatova E. Starks A. Goswami N. Allen L. Winston C. (2022). Interim guidance: 4-month rifapentine-moxifloxacin regimen for the treatment of drug-susceptible pulmonary tuberculosis - united states 2022. MMWR Morb Mortal Wkly Rep. 71 (8), 285–289. doi: 10.15585/mmwr.mm7108a1 35202353

[B13] Chandrasekaran S. Cokol-Cakmak M. Sahin N. Yilancioglu K. Kazan H. Collins J. J. . (2016). Chemogenomics and orthology-based design of antibiotic combination therapies. Mol. Syst. Biol. 12 (5), 872. doi: 10.15252/msb.20156777 27222539 PMC5289223

[B14] Chen Y. X. Xu Z. Y. Ge X. Sanyal S. Lu Z. J. Javid B. (2020). Selective translation by alternative bacterial ribosomes. Proc. Natl. Acad. Sci. U.S.A. 117 (32), 19487–19496. doi: 10.1073/pnas.2009607117 32723820 PMC7431078

[B15] Cho S. H. Warit S. Wan B. Hwang C. H. Pauli G. F. Franzblau S. G. (2007). Low-oxygen-recovery assay for high-throughput screening of compounds against nonreplicating mycobacterium tuberculosis. Antimicrob. Agents Chemother. 51 (4), 1380–1385. doi: 10.1128/AAC.00055-06 17210775 PMC1855511

[B16] Cicchese J. M. Sambarey A. Kirschner D. Linderman J. J. Chandrasekaran S. (2021). A multi-scale pipeline linking drug transcriptomics with pharmacokinetics predicts *in vivo* interactions of tuberculosis drugs. Sci. Rep. 11 (1), 5643. doi: 10.1038/s41598-021-84827-0 33707554 PMC7971003

[B17] Cokol M. Kuru N. Bicak E. Larkins-Ford J. Aldridge B. B. (2017). Efficient measurement and factorization of high-order drug interactions in mycobacterium tuberculosis. Sci. Adv. 3 (10), e1701881. doi: 10.1126/sciadv.1701881 29026882 PMC5636204

[B18] Conradie F. Diacon A. H. Ngubane N. Howell P. Everitt D. Crook A. M. . (2020). Treatment of highly drug-resistant pulmonary tuberculosis. N Engl. J. Med. 382 (10), 893–902. doi: 10.1056/NEJMoa1901814 32130813 PMC6955640

[B19] Daniel J. Maamar H. Deb C. Sirakova T. D. Kolattukudy P. E. (2011). Mycobacterium tuberculosis uses host triacylglycerol to accumulate lipid droplets and acquires a dormancy-like phenotype in lipid-loaded macrophages. PLoS Pathog. 7 (6), e1002093. doi: 10.1371/journal.ppat.1002093 21731490 PMC3121879

[B20] Deb C. Lee C. M. Dubey V. S. Daniel J. Abomoelak B. Sirakova T. D. . (2009). A novel *in vitro* multiple-stress dormancy model for mycobacterium tuberculosis generates a lipid-loaded, drug-tolerant, dormant pathogen. PLoS One 4 (6), e6077. doi: 10.1371/journal.pone.0006077 19562030 PMC2698117

[B21] De Groote M. A. Gruppo V. Woolhiser L. K. Orme I. M. Gilliland J. C. Lenaerts A. J. (2012). Importance of confirming data on the *in vivo* efficacy of novel antibacterial drug regimens against various strains of mycobacterium tuberculosis. Antimicrob. Agents Chemother. 56 (2), 731–738. doi: 10.1128/AAC.05701-11 22143517 PMC3264252

[B22] de Miranda Silva C. Hajihosseini A. Myrick J. Nole J. Louie A. Schmidt S. . (2019). Effect of moxifloxacin plus pretomanid against mycobacterium tuberculosis in log phase, acid phase, and nonreplicating-persister phase in an *In vitro* assay. Antimicrob. Agents Chemother. 63 (1). doi: 10.1128/AAC.01695-18 PMC6325209 30397058

[B23] Dickinson J. M. Mitchison D. A. (1976). Bactericidal activity *in vitro* and in the guinea-pig of isoniazid, rifampicin and ethambutol. Tubercle 57 (4), 251–258. doi: 10.1016/s0041-3879(76)80002-5 827836

[B24] Dickinson J. M. Mitchison D. A. (1981). Experimental models to explain the high sterilizing activity of rifampin in the chemotherapy of tuberculosis. Am. Rev. Respir. Dis. 123 (4 Pt 1), 367–371. doi: 10.1164/arrd.1981.123.4.367 6784622

[B25] Dide-Agossou C. Bauman A. A. Ramey M. E. Rossmassler K. Al Mubarak R. Pauly S. . (2022). Combination of mycobacterium tuberculosis RS ratio and CFU improves the ability of murine efficacy experiments to distinguish between drug treatments. Antimicrob. Agents Chemother. 66 (4), e0231021. doi: 10.1128/aac.02310-21 35311519 PMC9017352

[B26] Dorman S. E. Nahid P. Kurbatova E. V. Phillips P. P. J. Bryant K. Dooley K. E. . (2021). Four-month rifapentine regimens with or without moxifloxacin for tuberculosis. N Engl. J. Med. 384 (18), 1705–1718. doi: 10.1056/NEJMoa2033400 33951360 PMC8282329

[B27] Dow A. Sule P. O'Donnell T. J. Burger A. Mattila J. T. Antonio B. . (2021). Zinc limitation triggers anticipatory adaptations in mycobacterium tuberculosis. PloS Pathog. 17 (5), e1009570. doi: 10.1371/journal.ppat.1009570 33989345 PMC8121289

[B28] Driver E. R. Ryan G. J. Hoff D. R. Irwin S. M. Basaraba R. J. Kramnik I. . (2012). Evaluation of a mouse model of necrotic granuloma formation using C3HeB/FeJ mice for testing of drugs against mycobacterium tuberculosis. Antimicrob. Agents Chemother. 56 (6), 3181–3195. doi: 10.1128/AAC.00217-12 22470120 PMC3370740

[B29] Early J. V. Casey A. Martinez-Grau M. A. Gonzalez Valcarcel I. C. Vieth M. Ollinger J. . (2016). Oxadiazoles have butyrate-specific conditional activity against mycobacterium tuberculosis. Antimicrob. Agents Chemother. 60 (6), 3608–3616. doi: 10.1128/AAC.02896-15 27044545 PMC4879361

[B30] Early J. V. Mullen S. Parish T. (2019b). A rapid, low pH, nutrient stress, assay to determine the bactericidal activity of compounds against non-replicating mycobacterium tuberculosis. PloS One 14 (10), e0222970. doi: 10.1371/journal.pone.0222970 31589621 PMC6779252

[B31] Early J. Ollinger J. Darby C. Alling T. Mullen S. Casey A. . (2019a). Identification of compounds with pH-dependent bactericidal activity against mycobacterium tuberculosis. ACS Infect. Dis. 5 (2), 272–280. doi: 10.1021/acsinfecdis.8b00256 30501173 PMC6371205

[B32] East African- British medical research councils (1973). Controlled clinical trial of four short-course (6-month) regimens of chemotherapy for treatment of pulmonary tuberculosis. second report. Lancet 1 (7816), 1331–1338. 4122738

[B33] East African- British medical research councils (1974). Controlled clinical trial of four short-course (6-month) regimens of chemotherapy for treatment of pulmonary tuberculosis. third report. East African-British medical research councils. Lancet 2 (7875), 237–240. doi: 10.1016/S0140-6736(74)91411-1 4135686

[B34] Ernest J. P. Strydom N. Wang Q. Zhang N. Nuermberger E. Dartois V. . (2021). Development of new tuberculosis drugs: Translation to regimen composition for drug-sensitive and multidrug-resistant tuberculosis. Annu. Rev. Pharmacol. Toxicol. 61, 495–516. doi: 10.1146/annurev-pharmtox-030920-011143 32806997 PMC7790895

[B35] Fallahi-Sichani M. El-Kebir M. Marino S. Kirschner D. E. Linderman J. J. (2011). Multiscale computational modeling reveals a critical role for TNF-alpha receptor 1 dynamics in tuberculosis granuloma formation. J. Immunol. 186 (6), 3472–3483. doi: 10.4049/jimmunol.1003299 21321109 PMC3127549

[B36] Fors J. Strydom N. Fox W. S. Keizer R. J. Savic R. M. (2020). Mathematical model and tool to explore shorter multi-drug therapy options for active pulmonary tuberculosis. PLoS Comput. Biol. 16 (8), e1008107. doi: 10.1371/journal.pcbi.1008107 32810158 PMC7480878

[B37] Fox W. Ellard G. A. Mitchison D. A. (1999). Studies on the treatment of tuberculosis undertaken by the British medical research council tuberculosis units 1946-1986, with relevant subsequent publications. Int. J. Tuberc Lung Dis. 3 (10 Suppl 2), S231–S279. 10529902

[B38] Gold B. Roberts J. Ling Y. Quezada L. L. Glasheen J. Ballinger E. . (2015). Rapid, semiquantitative assay to discriminate among compounds with activity against replicating or nonreplicating mycobacterium tuberculosis. Antimicrob. Agents Chemother. 59 (10), 6521–6538. doi: 10.1128/AAC.00803-15 26239979 PMC4576094

[B39] Gouzy A. Healy C. Black K. A. Rhee K. Y. Ehrt S. (2021). Growth of mycobacterium tuberculosis at acidic pH depends on lipid assimilation and is accompanied by reduced GAPDH activity. Proc. Natl. Acad. Sci. U.S.A. 118 (32). doi: 10.1073/pnas.2024571118 PMC8364206 34341117

[B40] Grant S. S. Kawate T. Nag P. P. Silvis M. R. Gordon K. Stanley S. A. . (2013). Identification of novel inhibitors of nonreplicating mycobacterium tuberculosis using a carbon starvation model. ACS Chem. Biol. 8 (10), 2224–2234. doi: 10.1021/cb4004817 23898841 PMC3864639

[B41] Greenwood D. J. Dos Santos M. S. Huang S. Russell M. R. G. Collinson L. M. MacRae J. I. . (2019). Subcellular antibiotic visualization reveals a dynamic drug reservoir in infected macrophages. Science 364 (6447), 1279–1282. doi: 10.1126/science.aat9689 31249058 PMC7012645

[B42] Guerrini V. Prideaux B. Blanc L. Bruiners N. Arrigucci R. Singh S. . (2018). Storage lipid studies in tuberculosis reveal that foam cell biogenesis is disease-specific. PloS Pathog. 14 (8), e1007223. doi: 10.1371/journal.ppat.1007223 30161232 PMC6117085

[B43] Gumbo T. Louie A. Deziel M. R. Parsons L. M. Salfinger M. Drusano G. L. (2004). Selection of a moxifloxacin dose that suppresses drug resistance in mycobacterium tuberculosis, by use of an *in vitro* pharmacodynamic infection model and mathematical modeling. J. Infect. Dis. 190 (9), 1642–1651. doi: 10.1086/424849 15478070

[B44] Gumbo T. Pasipanodya J. G. Romero K. Hanna D. Nuermberger E. (2015). Forecasting accuracy of the hollow fiber model of tuberculosis for clinical therapeutic outcomes. Clin. Infect. Dis. 61 Suppl 1, S25–S31. doi: 10.1093/cid/civ427 26224769

[B45] Harper J. Skerry C. Davis S. L. Tasneen R. Weir M. Kramnik I. . (2012). Mouse model of necrotic tuberculosis granulomas develops hypoxic lesions. J. Infect. Dis. 205 (4), 595–602. doi: 10.1093/infdis/jir786 22198962 PMC3266133

[B46] Hicks N. D. Yang J. Zhang X. Zhao B. Grad Y. H. Liu L. . (2018). Clinically prevalent mutations in mycobacterium tuberculosis alter propionate metabolism and mediate multidrug tolerance. Nat. Microbiol. 3 (9), 1032–1042. doi: 10.1038/s41564-018-0218-3 30082724 PMC6233875

[B47] Hu Y. Coates A. R. Mitchison D. A. (2006). Sterilising action of pyrazinamide in models of dormant and rifampicin-tolerant mycobacterium tuberculosis. Int. J. Tuberc Lung Dis. 10 (3), 317–322. 16562713

[B48] Irwin S. M. Gruppo V. Brooks E. Gilliland J. Scherman M. Reichlen M. J. . (2014). Limited activity of clofazimine as a single drug in a mouse model of tuberculosis exhibiting caseous necrotic granulomas. Antimicrob. Agents Chemother. 58 (7), 4026–4034. doi: 10.1128/AAC.02565-14 24798275 PMC4068578

[B49] Jones R. M. Adams K. N. Eldesouky H. E. Sherman D. R. (2022). The evolving biology of mycobacterium tuberculosis drug resistance. Front. Cell Infect. Microbiol. 12. doi: 10.3389/fcimb.2022.1027394 PMC9579286 36275024

[B50] Katzir I. Cokol M. Aldridge B. B. Alon U. (2019). Prediction of ultra-high-order antibiotic combinations based on pairwise interactions. PloS Comput. Biol. 15 (1), e1006774. doi: 10.1371/journal.pcbi.1006774 30699106 PMC6370231

[B51] Kempker R. R. Heinrichs M. T. Nikolaishvili K. Sabulua I. Bablishvili N. Gogishvili S. . (2017). Lung tissue concentrations of pyrazinamide among patients with drug-resistant pulmonary tuberculosis. Antimicrob. Agents Chemother. 61 (6). doi: 10.1128/AAC.00226-17 PMC5444116 28373198

[B52] Kerantzas C. A. Jacobs W. R. Jr (2017). Origins of combination therapy for tuberculosis: Lessons for future antimicrobial development and application. mBio 8 (2). doi: 10.1128/mBio.01586-16 PMC5350467 28292983

[B53] Kjellsson M. C. Via L. E. Goh A. Weiner D. Low K. M. Kern S. . (2012). Pharmacokinetic evaluation of the penetration of antituberculosis agents in rabbit pulmonary lesions. Antimicrob. Agents Chemother. 56 (1), 446–457. doi: 10.1128/AAC.05208-11 21986820 PMC3256032

[B54] Koul A. Vranckx L. Dendouga N. Balemans W. Van den Wyngaert I. Vergauwen K. . (2008). Diarylquinolines are bactericidal for dormant mycobacteria as a result of disturbed ATP homeostasis. J. Biol. Chem. 283 (37), 25273–25280. doi: 10.1074/jbc.M803899200 18625705

[B55] Kurthkoti K. Amin H. Marakalala M. J. Ghanny S. Subbian S. Sakatos A. . (2017). The capacity of mycobacterium tuberculosis to survive iron starvation might enable it to persist in iron-deprived microenvironments of human granulomas. mBio 8 (4). doi: 10.1128/mBio.01092-17 PMC5559634 28811344

[B56] Lanoix J. P. Ioerger T. Ormond A. Kaya F. Sacchettini J. Dartois V. . (2016). Selective inactivity of pyrazinamide against tuberculosis in C3HeB/FeJ mice is best explained by neutral pH of caseum. Antimicrob. Agents Chemother. 60 (2), 735–743. doi: 10.1128/AAC.01370-15 26574016 PMC4750710

[B57] Larkins-Ford J. Degefu Y. N. Van N. Sokolov A. Aldridge B. B. (2022). Design principles to assemble drug combinations for effective tuberculosis therapy using interpretable pairwise drug response measurements. Cell Rep. Med. 3 (9), 100737. doi: 10.1016/j.xcrm.2022.100737 36084643 PMC9512659

[B58] Larkins-Ford J. Greenstein T. Van N. Degefu Y. N. Olson M. C. Sokolov A. . (2021). Systematic measurement of combination-drug landscapes to predict *in vivo* treatment outcomes for tuberculosis. Cell Syst. 12 (11), 1046–1063.e1047. doi: 10.1016/j.cels.2021.08.004 34469743 PMC8617591

[B59] Lavin R. C. Tan S. (2022). Spatial relationships of intra-lesion heterogeneity in mycobacterium tuberculosis microenvironment, replication status, and drug efficacy. PloS Pathog. 18 (3), e1010459. doi: 10.1371/journal.ppat.1010459 35344572 PMC8989358

[B60] Lee W. VanderVen B. C. Fahey R. J. Russell D. G. (2013). Intracellular mycobacterium tuberculosis exploits host-derived fatty acids to limit metabolic stress. J. Biol. Chem. 288 (10), 6788–6800. doi: 10.1074/jbc.M112.445056 23306194 PMC3591590

[B61] Lenaerts A. Barry C. E. 3rd Dartois V. (2015). Heterogeneity in tuberculosis pathology, microenvironments and therapeutic responses. Immunol. Rev. 264 (1), 288–307. doi: 10.1111/imr.12252 25703567 PMC4368385

[B62] Lenaerts A. J. Hoff D. Aly S. Ehlers S. Andries K. Cantarero L. . (2007). Location of persisting mycobacteria in a Guinea pig model of tuberculosis revealed by r207910. Antimicrob. Agents Chemother. 51 (9), 3338–3345. doi: 10.1128/AAC.00276-07 17517834 PMC2043239

[B63] Liebenberg D. Gordhan B. G. Kana B. D. (2022). Drug resistant tuberculosis: Implications for transmission, diagnosis, and disease management. Front. Cell Infect. Microbiol. 12. doi: 10.3389/fcimb.2022.943545 PMC9538507 36211964

[B64] Li S. Poulton N. C. Chang J. S. Azadian Z. A. DeJesus M. A. Ruecker N. . (2022). CRISPRi chemical genetics and comparative genomics identify genes mediating drug potency in mycobacterium tuberculosis. Nat. Microbiol. 7 (6), 766–779. doi: 10.1038/s41564-022-01130-y 35637331 PMC9159947

[B65] Liu Q. Zhu J. Dulberger C. L. Stanley S. Wilson S. Chung E. S. . (2022). Tuberculosis treatment failure associated with evolution of antibiotic resilience. Science 378 (6624), 1111–1118. doi: 10.1126/science.abq2787 36480634 PMC9968493

[B66] Lyons M. A. (2022). Pharmacodynamics and bactericidal activity of bedaquiline in pulmonary tuberculosis. Antimicrob. Agents Chemother. 66 (2), e0163621. doi: 10.1128/AAC.01636-21 34871099 PMC8846494

[B67] MacGilvary N. J. Kevorkian Y. L. Tan S. (2019). Potassium response and homeostasis in mycobacterium tuberculosis modulates environmental adaptation and is important for host colonization. PLoS Pathog. 15 (2), e1007591. doi: 10.1371/journal.ppat.1007591 30716121 PMC6375644

[B68] Ma S. Jaipalli S. Larkins-Ford J. Lohmiller J. Aldridge B. B. Sherman D. R. . (2019). Transcriptomic signatures predict regulators of drug synergy and clinical regimen efficacy against tuberculosis. mBio 10 (6). doi: 10.1128/mBio.02627-19 PMC6851285 31719182

[B69] Manina G. Dhar N. McKinney J. D. (2015). Stress and host immunity amplify mycobacterium tuberculosis phenotypic heterogeneity and induce nongrowing metabolically active forms. Cell Host Microbe 17 (1), 32–46. doi: 10.1016/j.chom.2014.11.016 25543231

[B70] Marrero J. Rhee K. Y. Schnappinger D. Pethe K. Ehrt S. (2010). Gluconeogenic carbon flow of tricarboxylic acid cycle intermediates is critical for mycobacterium tuberculosis to establish and maintain infection. Proc. Natl. Acad. Sci. U.S.A. 107 (21), 9819–9824. doi: 10.1073/pnas.1000715107 20439709 PMC2906907

[B71] Muttucumaru D. G. Roberts G. Hinds J. Stabler R. A. Parish T. (2004). Gene expression profile of mycobacterium tuberculosis in a non-replicating state. Tuberculosis (Edinb) 84 (3-4), 239–246. doi: 10.1016/j.tube.2003.12.006 15207493

[B72] Neyrolles O. Wolschendorf F. Mitra A. Niederweis M. (2015). Mycobacteria, metals, and the macrophage. Immunol. Rev. 264 (1), 249–263. doi: 10.1111/imr.12265 25703564 PMC4521620

[B73] Nuermberger E. L. Yoshimatsu T. Tyagi S. O'Brien R. J. Vernon A. N. Chaisson R. E. . (2004). Moxifloxacin-containing regimen greatly reduces time to culture conversion in murine tuberculosis. Am. J. Respir. Crit. Care Med. 169 (3), 421–426. doi: 10.1164/rccm.200310-1380OC 14578218

[B74] Pandey A. K. Sassetti C. M. (2008). Mycobacterial persistence requires the utilization of host cholesterol. Proc. Natl. Acad. Sci. U.S.A. 105 (11), 4376–4380. doi: 10.1073/pnas.0711159105 18334639 PMC2393810

[B75] Peterson E. J. R. Ma S. Sherman D. R. Baliga N. S. (2016). Network analysis identifies Rv0324 and Rv0880 as regulators of bedaquiline tolerance in mycobacterium tuberculosis. Nat. Microbiol. 1 (8), 16078. doi: 10.1038/nmicrobiol.2016.78 27573104 PMC5010021

[B76] Pethe K. Sequeira P. C. Agarwalla S. Rhee K. Kuhen K. Phong W. Y. . (2010). A chemical genetic screen in mycobacterium tuberculosis identifies carbon-source-dependent growth inhibitors devoid of *in vivo* efficacy. Nat. Commun. 1, 57. doi: 10.1038/ncomms1060 20975714 PMC3220188

[B77] Peyron P. Vaubourgeix J. Poquet Y. Levillain F. Botanch C. Bardou F. . (2008). Foamy macrophages from tuberculous patients' granulomas constitute a nutrient-rich reservoir for m. tuberculosis persistence. PLoS Pathog. 4 (11), e1000204. doi: 10.1371/journal.ppat.1000204 19002241 PMC2575403

[B78] Piddington D. L. Kashkouli A. Buchmeier N. A. (2000). Growth of mycobacterium tuberculosis in a defined medium is very restricted by acid pH and Mg(2+) levels. Infect. Immun. 68 (8), 4518–4522. doi: 10.1128/IAI.68.8.4518-4522.2000 10899850 PMC98362

[B79] Pienaar E. Matern W. M. Linderman J. J. Bader J. S. Kirschner D. E. (2016). Multiscale model of mycobacterium tuberculosis infection maps metabolite and gene perturbations to granuloma sterilization predictions. Infect. Immun. 84 (5), 1650–1669. doi: 10.1128/IAI.01438-15 26975995 PMC4862722

[B80] Pienaar E. Sarathy J. Prideaux B. Dietzold J. Dartois V. Kirschner D. E. . (2017). Comparing efficacies of moxifloxacin, levofloxacin and gatifloxacin in tuberculosis granulomas using a multi-scale systems pharmacology approach. PloS Comput. Biol. 13 (8), e1005650. doi: 10.1371/journal.pcbi.1005650 28817561 PMC5560534

[B81] Prideaux B. Via L. E. Zimmerman M. D. Eum S. Sarathy J. O'Brien P. . (2015). The association between sterilizing activity and drug distribution into tuberculosis lesions. Nat. Med. 21 (10), 1223–1227. doi: 10.1038/nm.3937 26343800 PMC4598290

[B82] Prisic S. Hwang H. Dow A. Barnaby O. Pan T. S. Lonzanida J. A. . (2015). Zinc regulates a switch between primary and alternative S18 ribosomal proteins in mycobacterium tuberculosis. Mol. Microbiol. 97 (2), 263–280. doi: 10.1111/mmi.13022 25858183 PMC4548965

[B83] Quinonez C. G. Lee J. J. Lim J. Odell M. Lawson C. P. Anyogu A. . (2022). The role of fatty acid metabolism in drug tolerance of mycobacterium tuberculosis. mBio 13 (1), e0355921. doi: 10.1128/mbio.03559-21 35012349 PMC8749430

[B84] Rao S. P. Alonso S. Rand L. Dick T. Pethe K. (2008). The protonmotive force is required for maintaining ATP homeostasis and viability of hypoxic, nonreplicating mycobacterium tuberculosis. Proc. Natl. Acad. Sci. U.S.A. 105 (33), 11945–11950. doi: 10.1073/pnas.0711697105 18697942 PMC2575262

[B85] Rego E. H. Audette R. E. Rubin E. J. (2017). Deletion of a mycobacterial divisome factor collapses single-cell phenotypic heterogeneity. Nature 546 (7656), 153–157. doi: 10.1038/nature22361 28569798 PMC5567998

[B86] Rodriguez J. G. Hernandez A. C. Helguera-Repetto C. Aguilar Ayala D. Guadarrama-Medina R. Anzola J. M. . (2014). Global adaptation to a lipid environment triggers the dormancy-related phenotype of mycobacterium tuberculosis. mBio 5 (3), e01125–e01114. doi: 10.1128/mBio.01125-14 24846381 PMC4030484

[B87] Rodriguez G. M. Sharma N. Biswas A. Sharma N. (2022). The iron response of mycobacterium tuberculosis and its implications for tuberculosis pathogenesis and novel therapeutics. Front. Cell Infect. Microbiol. 12. doi: 10.3389/fcimb.2022.876667 PMC9132128 35646739

[B88] Russell D. G. Cardona P. J. Kim M. J. Allain S. Altare F. (2009). Foamy macrophages and the progression of the human tuberculosis granuloma. Nat. Immunol. 10 (9), 943–948. doi: 10.1038/ni.1781 19692995 PMC2759071

[B89] Sarathy J. P. Dartois V. (2020). Caseum: a niche for mycobacterium tuberculosis drug-tolerant persisters. Clin. Microbiol. Rev. 33 (3). doi: 10.1128/CMR.00159-19 PMC7117546 32238365

[B90] Sarathy J. P. Via L. E. Weiner D. Blanc L. Boshoff H. Eugenin E. A. . (2018). Extreme drug tolerance of mycobacterium tuberculosis in caseum. Antimicrob. Agents Chemother. 62 (2). doi: 10.1128/AAC.02266-17 PMC5786764 29203492

[B91] Sarathy J. P. Zuccotto F. Hsinpin H. Sandberg L. Via L. E. Marriner G. A. . (2016). Prediction of drug penetration in tuberculosis lesions. ACS Infect. Dis. 2 (8), 552–563. doi: 10.1021/acsinfecdis.6b00051 27626295 PMC5028112

[B92] Savic R. M. Weiner M. MacKenzie W. R. Engle M. Whitworth W. C. Johnson J. L. . (2017). Defining the optimal dose of rifapentine for pulmonary tuberculosis: Exposure-response relations from two phase II clinical trials. Clin. Pharmacol. Ther. 102 (2), 321–331. doi: 10.1002/cpt.634 28124478 PMC5545752

[B93] Schnappinger D. Ehrt S. Voskuil M. I. Liu Y. Mangan J. A. Monahan I. M. . (2003). Transcriptional adaptation of mycobacterium tuberculosis within macrophages: Insights into the phagosomal environment. J. Exp. Med. 198 (5), 693–704. doi: 10.1084/jem.20030846 12953091 PMC2194186

[B94] Schrader S. M. Botella H. Jansen R. Ehrt S. Rhee K. Nathan C. . (2021). Multiform antimicrobial resistance from a metabolic mutation. Sci. Adv. 7 (35). doi: 10.1126/sciadv.abh2037 PMC8397267 34452915

[B95] Shee S. Singh S. Tripathi A. Thakur C. Kumar T. A. Das M. . (2022). Moxifloxacin-mediated killing of mycobacterium tuberculosis involves respiratory downshift, reductive stress, and accumulation of reactive oxygen species. Antimicrob. Agents Chemother. 66 (9), e0059222. doi: 10.1128/aac.00592-22 35975988 PMC9487606

[B96] Sherman D. R. Voskuil M. Schnappinger D. Liao R. Harrell M. I. Schoolnik G. K. (2001). Regulation of the mycobacterium tuberculosis hypoxic response gene encoding alpha -crystallin. Proc. Natl. Acad. Sci. U.S.A. 98 (13), 7534–7539. doi: 10.1073/pnas.121172498 11416222 PMC34703

[B97] Sohaskey C. D. (2008). Nitrate enhances the survival of mycobacterium tuberculosis during inhibition of respiration. J. Bacteriol. 190 (8), 2981–2986. doi: 10.1128/JB.01857-07 18296525 PMC2293237

[B98] Soto-Ramirez M. D. Aguilar-Ayala D. A. Garcia-Morales L. Rodriguez-Peredo S. M. Badillo-Lopez C. Rios-Muniz D. E. . (2017). Cholesterol plays a larger role during mycobacterium tuberculosis *in vitro* dormancy and reactivation than previously suspected. Tuberculosis (Edinb) 103, 1–9. doi: 10.1016/j.tube.2016.12.004 28237027

[B99] Steenken W. Jr. Wolinsky E. (1954). The antituberculous activity of pyrazinamide *in vitro* and in the guinea pig. Am. Rev. Tuberc 70 (2), 367–369. doi: 10.1164/art.1954.70.2.367 13180876

[B100] Strydom N. Gupta S. V. Fox W. S. Via L. E. Bang H. Lee M. . (2019). Tuberculosis drugs' distribution and emergence of resistance in patient's lung lesions: A mechanistic model and tool for regimen and dose optimization. PloS Med. 16 (4), e1002773. doi: 10.1371/journal.pmed.1002773 30939136 PMC6445413

[B101] Tan S. Sukumar N. Abramovitch R. B. Parish T. Russell D. G. (2013). Mycobacterium tuberculosis responds to chloride and pH as synergistic cues to the immune status of its host cell. PloS Pathog. 9 (4), e1003282. doi: 10.1371/journal.ppat.1003282 23592993 PMC3616970

[B102] Tasneen R. Li S. Y. Peloquin C. A. Taylor D. Williams K. N. Andries K. . (2011). Sterilizing activity of novel TMC207- and PA-824-containing regimens in a murine model of tuberculosis. Antimicrob. Agents Chemother. 55 (12), 5485–5492. doi: 10.1128/AAC.05293-11 21930883 PMC3232786

[B103] Theriault M. E. Pisu D. Wilburn K. M. Le-Bury G. MacNamara C. W. Michael Petrassi H. . (2022). Iron limitation in m. tuberculosis has broad impact on central carbon metabolism. Commun. Biol. 5 (1), 685. doi: 10.1038/s42003-022-03650-z 35810253 PMC9271047

[B104] Tizzano B. Dallenga T. K. Utpatel C. Behrends J. Homolka S. Kohl T. A. . (2021). Survival of hypoxia-induced dormancy is not a common feature of all strains of the mycobacterium tuberculosis complex. Sci. Rep. 11 (1), 2628. doi: 10.1038/s41598-021-81223-6 33514768 PMC7846770

[B105] Trutneva K. A. Shleeva M. O. Demina G. R. Vostroknutova G. N. Kaprelyans A. S. (2020). One-year old dormant, "Non-culturable" mycobacterium tuberculosis preserves significantly diverse protein profile. Front. Cell Infect. Microbiol. 10. doi: 10.3389/fcimb.2020.00026 PMC7025520 32117801

[B106] Via L. E. England K. Weiner D. M. Schimel D. Zimmerman M. D. Dayao E. . (2015). A sterilizing tuberculosis treatment regimen is associated with faster clearance of bacteria in cavitary lesions in marmosets. Antimicrob. Agents Chemother. 59 (7), 4181–4189. doi: 10.1128/AAC.00115-15 25941223 PMC4468655

[B107] Via L. E. Lin P. L. Ray S. M. Carrillo J. Allen S. S. Eum S. Y. . (2008). Tuberculous granulomas are hypoxic in guinea pigs, rabbits, and nonhuman primates. Infect. Immun. 76 (6), 2333–2340. doi: 10.1128/IAI.01515-07 18347040 PMC2423064

[B108] Wakamoto Y. Dhar N. Chait R. Schneider K. Signorino-Gelo F. Leibler S. . (2013). Dynamic persistence of antibiotic-stressed mycobacteria. Science 339 (6115), 91–95. doi: 10.1126/science.1229858 23288538

[B109] Walter N. D. Born S. E. M. Robertson G. T. Reichlen M. Dide-Agossou C. Ektnitphong V. A. . (2021). Mycobacterium tuberculosis precursor rRNA as a measure of treatment-shortening activity of drugs and regimens. Nat. Commun. 12 (1), 2899. doi: 10.1038/s41467-021-22833-6 34006838 PMC8131613

[B110] Wayne L. G. Hayes L. G. (1996). An *in vitro* model for sequential study of shiftdown of mycobacterium tuberculosis through two stages of nonreplicating persistence. Infect. Immun. 64 (6), 2062–2069. doi: 10.1128/iai.64.6.2062-2069.1996 8675308 PMC174037

[B111] WHO (2021). Global tuberculosis report 2021 (Geneva: World Health Organization).

[B112] Wilburn K. M. Fieweger R. A. VanderVen B. C. (2018). Cholesterol and fatty acids grease the wheels of mycobacterium tuberculosis pathogenesis. Pathog. Dis. 76 (2). doi: 10.1093/femspd/fty021 PMC6251666 29718271

[B113] Wong E. A. Evans S. Kraus C. R. Engelman K. D. Maiello P. Flores W. J. . (2020). IL-10 impairs local immune response in lung granulomas and lymph nodes during early mycobacterium tuberculosis infection. J. Immunol. 204 (3), 644–659. doi: 10.4049/jimmunol.1901211 31862711 PMC6981067

[B114] Wooten D. J. Meyer C. T. Lubbock A. L. R. Quaranta V. Lopez C. F. (2021). MuSyC is a consensus framework that unifies multi-drug synergy metrics for combinatorial drug discovery. Nat. Commun. 12 (1), 4607. doi: 10.1038/s41467-021-24789-z 34326325 PMC8322415

[B115] Xie Y. L. de Jager V. R. Chen R. Y. Dodd L. E. Paripati P. Via L. E. . (2021). Fourteen-day PET/CT imaging to monitor drug combination activity in treated individuals with tuberculosis. Sci. Transl. Med. 13 (579). doi: 10.1126/scitranslmed.abd7618 PMC11135015 33536283

[B116] Zheng H. Colvin C. J. Johnson B. K. Kirchhoff P. D. Wilson M. Jorgensen-Muga K. . (2017). Inhibitors of mycobacterium tuberculosis DosRST signaling and persistence. Nat. Chem. Biol. 13 (2), 218–225. doi: 10.1038/nchembio.2259 27992879

[B117] Zhu J. H. Wang B. W. Pan M. Zeng Y. N. Rego H. Javid B. (2018). Rifampicin can induce antibiotic tolerance in mycobacteria *via* paradoxical changes in rpoB transcription. Nat. Commun. 9 (1), 4218. doi: 10.1038/s41467-018-06667-3 30310059 PMC6181997

[B118] Zimmer A. Katzir I. Dekel E. Mayo A. E. Alon U. (2016). Prediction of multidimensional drug dose responses based on measurements of drug pairs. Proc. Natl. Acad. Sci. U.S.A. 113 (37), 10442–10447. doi: 10.1073/pnas.1606301113 27562164 PMC5027409

